# Quantification of NLRP3–caspase-1 complex assembly reveals a non-linear association with 30-day mortality in sepsis: a proof-of-concept pilot study

**DOI:** 10.1186/s40635-026-00992-4

**Published:** 2026-10-29

**Authors:** Fabian Mehl, Willy J. Chedjou, Andrea Witowski, Katharina Rump, Birte Dyck, Lars Bergmann, Christian Putensen, Dietrich Henzler, Michael Adamzik, Björn Koos, Patrick Thon, Tim Rahmel

**Affiliations:** 1 Ruhr-Universität Bochum; Klinik für Anästhesiologie, Intensivmedizin und Schmerztherapie; Knappschaft Kliniken - Universitätsklinikum Bochum, In der Schornau 23-25, Bochum, 44892 Germany; 2 Klinik für Anästhesiologie und Operative Intensivmedizin, Universitätsklinikum Bonn, Bonn, 53127 Germany; 3 Department of Anesthesiology, Surgical Intensive Care, Emergency and Pain Medicine, Klinikum Herford, Ruhr University Bochum, Herford, 32049 Germany

**Keywords:** Sepsis, NLRP3 inflammasome, Inflammasome activation, Proximity ligation assay, Immune phenotyping, Mortality

## Abstract

**Background:**

Direct, cell-resolved quantification of NLRP3 inflammasome activation in clinical patients has not been feasible. Previous studies have relied on indirect surrogates such as downstream cytokines, which are influenced by inflammasome-independent pathways and systemic clearance kinetics, potentially explaining the inconsistent prognostic findings reported in the literature. We applied a proximity ligation assay (PLA) to directly visualise NLRP3-caspase-1 complex formation in peripheral blood mononuclear cells of septic patients, providing a cell-specific, in situ readout of inflammasome assembly at the single-cell level.

**Methods:**

In this prospective, multicentre proof-of-concept pilot study, 78 patients fulfilling Sepsis-3 criteria were enrolled across three university hospitals. The PLA was validated in THP-1-derived macrophages against established readouts of canonical inflammasome activation prior to clinical application. Patients were stratified into low (n = 28), intermediate (n = 23), and high (n = 27) NLRP3 inflammasome activity groups.

**Results:**

Median age was 64 years (IQR: 56-78), 63% were male, and median Sequential Organ Failure Assessment score was 8 (IQR: 4-12). Baseline characteristics, disease severity scores, and routine inflammatory biomarkers did not differ across activity groups. Kaplan-Meier analysis revealed a non-linear pattern of 30-day survival, with the most favourable outcomes in the intermediate activity group. Both low and high activity were associated with increased mortality (log-rank p = 0.003). In adjusted Cox regression, low inflammasome activity (HR 7.88, 95% CI 1.79-34.65; p = 0.006) and high inflammasome activity (HR 4.35, 95% CI 1.01-19.88; p = 0.048) remained associated with 30-day mortality. Modelling PLA activity as a continuous variable using restricted cubic splines confirmed a significant non-linear association with 30-day mortality, independent of the tertile-based categorisation (p for non-linearity = 0.015).

**Conclusions:**

This proof-of-concept pilot study demonstrates that direct in situ quantification of NLRP3–caspase-1 complex assembly is feasible in septic patients and reveals a non-linear association with 30-day survival. These findings indicate that PLA-based profiling captures immune heterogeneity beyond routine inflammatory biomarkers. Larger independent cohorts are needed to validate these findings before clinical application or patient stratification can be considered.

**Supplementary Information:**

The online version contains supplementary material available at https://doi.org/10.1186/s40635-026-00992-4.

## Background

Sepsis is a life-threatening syndrome caused by a dysregulated host response to an infection [1]. A hallmark of this dysregulation is the profound imbalance between pro- and anti-inflammatory mechanisms, which may culminate in septic shock, immune paralysis, multiorgan failure and death [2, 3]. Importantly, sepsis is no longer viewed as a purely hyperinflammatory condition but rather as a dynamic and heterogeneous immune disorder in which excessive inflammation and immune suppression coexist or sequentially develop [4]. A deeper understanding of these immunological trajectories is essential for the development of reliable biomarkers and targeted therapeutic strategies.

A central yet incompletely understood component of this response is the inflammasome. Inflammasomes are cytosolic multiprotein complexes of the innate immune system that sense pathogen-associated and damage-associated molecular patterns [5]. Upon activation, NLRP3 recruits Apoptosis-associated speck-like protein containing a caspase recruitment domain (ASC) and pro-caspase-1, leading to caspase-1 cleavage, maturation of interleukin (IL)-1β and IL-18, and gasdermin D–mediated pyroptosis [6, 7]. Through these mechanisms, inflammasomes play a pivotal role in pathogen elimination and in orchestrating the early innate immune response.

While their activation is indispensable for effective host defence, both excessive and insufficient inflammasome activity may be detrimental and are associated with adverse clinical outcomes [8, 9]. Accordingly, accumulating evidence suggests that inflammasome activation in sepsis represents a biological “double-edged sword” [10]. On the one hand, excessive activation of NLRP3 has been associated with overwhelming systemic inflammation, endothelial dysfunction, disseminated intravascular coagulation, and mitochondrial injury, ultimately contributing to organ failure and increased mortality [6]. Inflammasome-driven IL-1β and IL-18 release can amplify cytokine cascades, promote oxidative stress, and induce mitochondrial dysfunction, which itself further fuels NLRP3 activation in a feed-forward loop. Such uncontrolled inflammasome signalling has been linked to adverse outcomes in experimental and clinical sepsis [11, 12].

On the other hand, insufficient or exhausted inflammasome activation may equally be detrimental. Sepsis-induced immunosuppression is characterized by impaired antigen presentation, reduced cytokine production, and functional reprogramming of innate immune cells [10]. Clinical studies have shown that lower levels of ASC-speck–positive monocytes during later phases of sepsis are associated with increased mortality [11, 13, 14], suggesting that inadequate inflammasome activation may reflect or contribute to immune paralysis and impaired pathogen control. Thus, both excessive and insufficient inflammasome activity may be associated with poor outcomes, pointing toward a potentially non-linear—possibly U-shaped—relationship between inflammasome activation and mortality.

Interpretation of existing clinical data, however, remains challenging. Many studies rely on circulating IL-1β and IL-18 levels as surrogate markers of inflammasome activity. Yet these cytokines can be induced by inflammasome-independent pathways, are influenced by clearance kinetics, receptor binding, and systemic distribution, and display short half-lives with substantial inter-individual variability [6]. Consequently, serum cytokine concentrations likely not reliably reflect cell-specific inflammasome activation with the necessary precision. Similarly, caspase-1 activity assays or detection of ASC-specks represent downstream or indirect markers and do not allow precise spatial and cell-specific quantification of NLRP3 inflammasome assembly [5, 14]. These methodological limitations may partly explain the heterogeneous and sometimes contradictory findings in the literature regarding the prognostic role of inflammasome activation in sepsis [10].

We therefore hypothesized that a direct, cell-specific, in situ functional assessment of NLRP3 inflammasome assembly could overcome these limitations. By applying a proximity ligation assay (PLA) to visualize NLRP3–caspase-1 complex formation in isolated peripheral blood mononuclear cells (PBMCs), inflammasome activation can be detected at the single-cell level, enabling spatial resolution and functional specificity beyond soluble cytokine measurements.

Accordingly, the primary objective of this proof-of-concept pilot study was to evaluate whether PLA-based inflammasome activation is associated with mortality in sepsis, following a non-linear (U-shaped) relationship, with both low and high activation levels conferring increased risk compared to intermediate levels.

## Results

### Patient characteristics

**Fig. 1:**
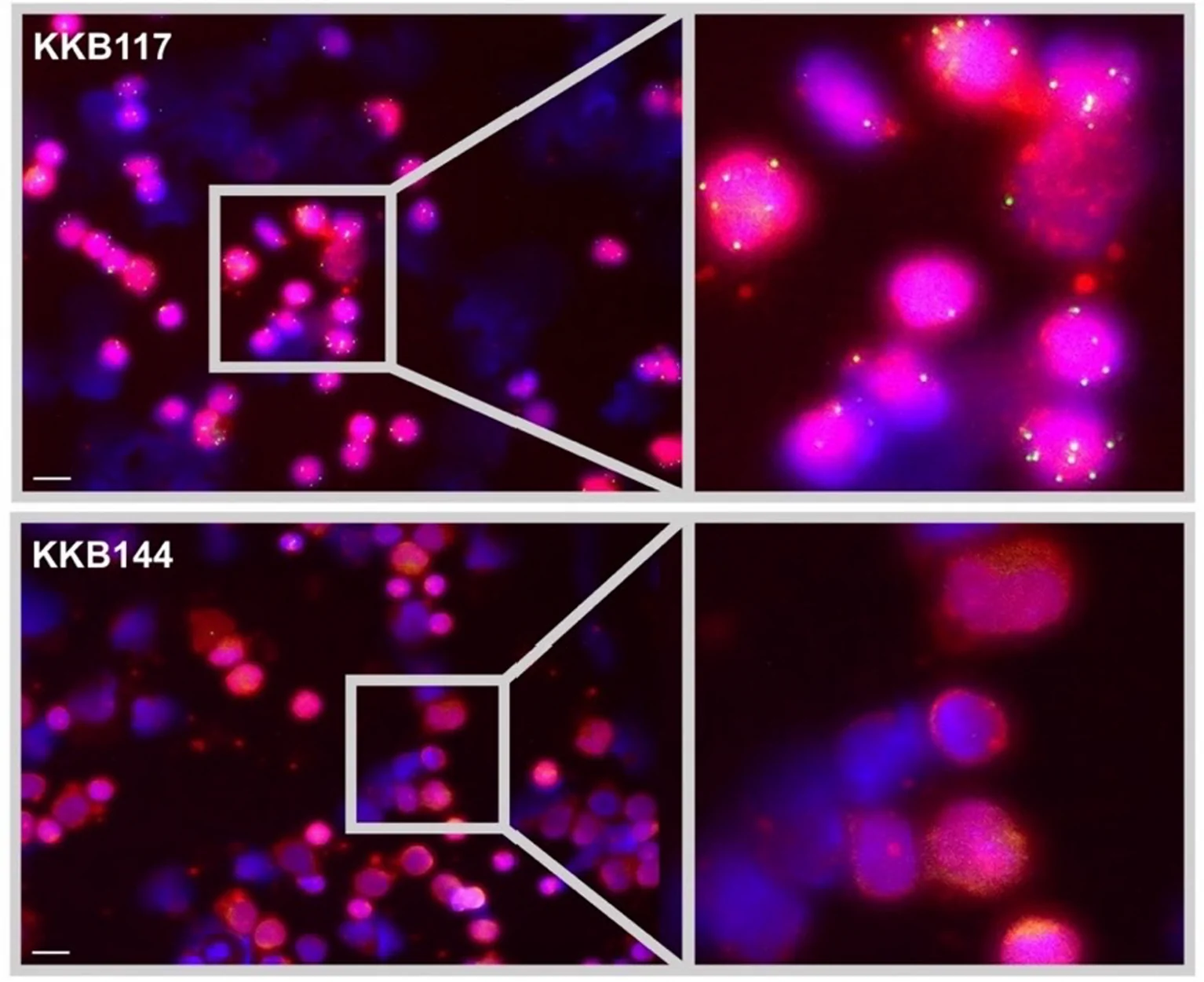
Representative immunofluorescence images illustrating the differential NLRP3 inflammasome activation in septic patients. Upper panel: High inflammasome activation with abundant NLRP3–Caspase-1 PLA signals. Lower panel: Low inflammasome activation with sparse signal formation. Nuclei (blue), PLA signals (green), β-actin (red). Scale bar = 10 µm

**Fig. 2:**
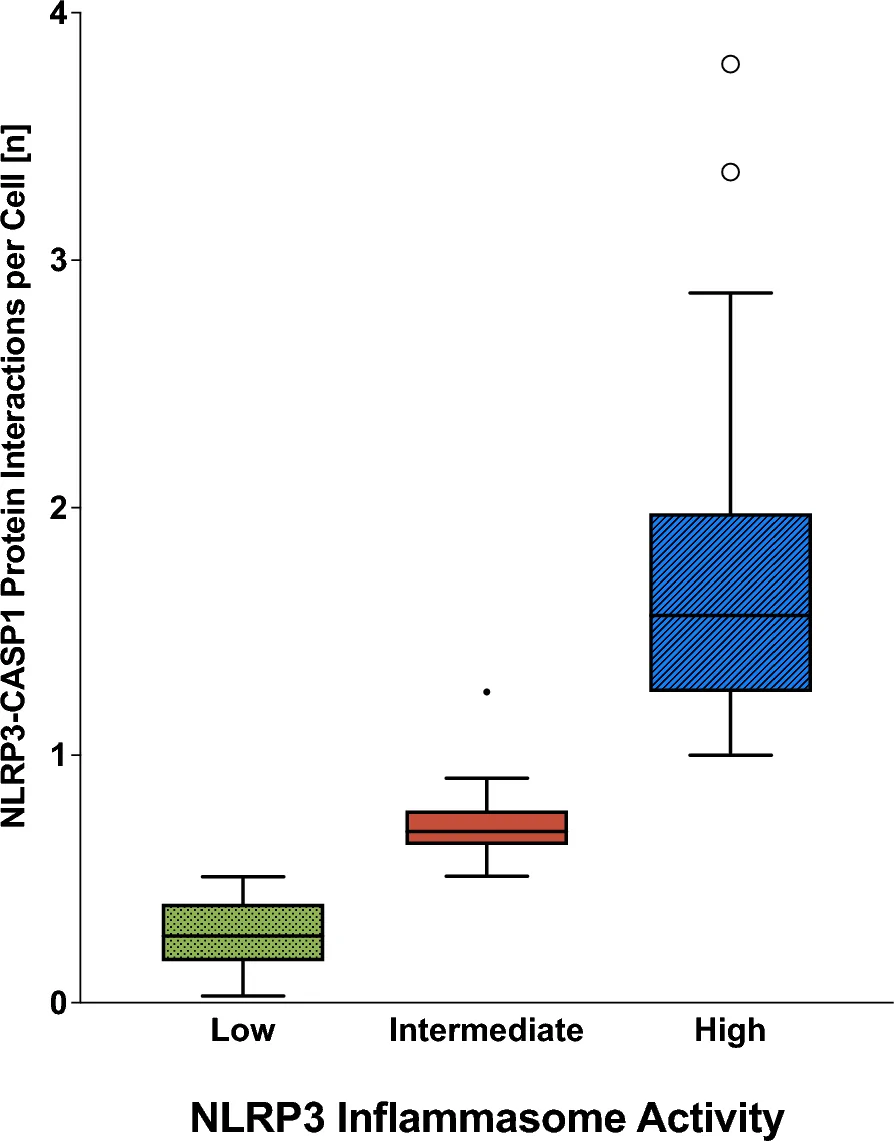
Distribution of NLRP3–Caspase-1 PLA interactions per cell across “low”, “intermediate”, and “high” inflammasome activation. Boxplots depict median and interquartile range. Overall group comparison by Kruskal–Wallis test (p < 0.001)

**Fig. 3:**
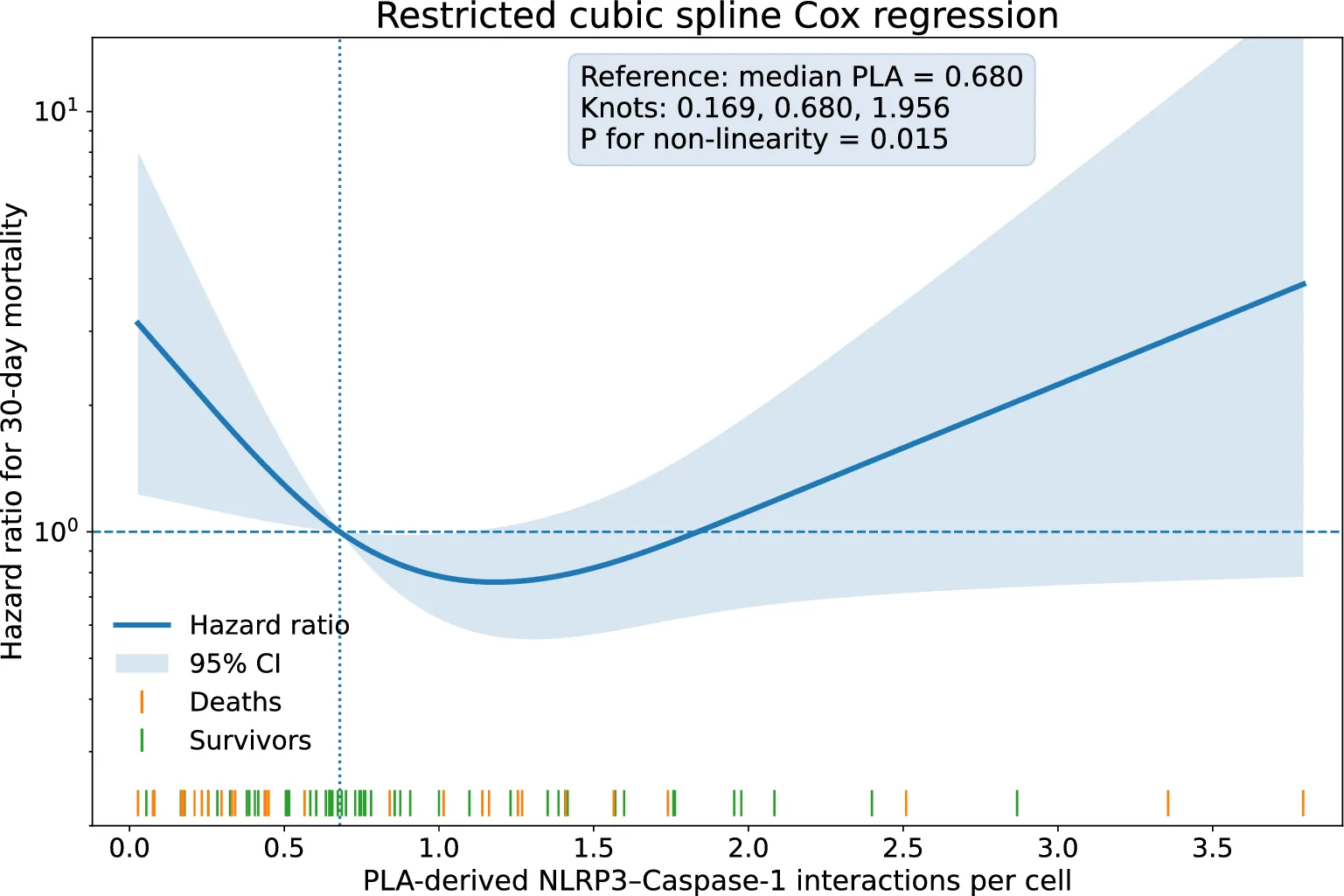
Restricted cubic spline analysis of PLA-derived NLRP3 inflammasome activity and 30-day mortality. PLA-derived NLRP3–caspase-1 interaction signals per cell were modelled as a continuous predictor of 30-day mortality in a Cox proportional hazards model using restricted cubic splines with three knots placed at the 10th, 50th and 90th percentiles of the PLA distribution. The median PLA value served as the reference (hazard ratio = 1, dashed horizontal line). The solid line indicates the estimated hazard ratio across the observed range of PLA activity, the shaded area the corresponding 95% confidence interval. The model was adjusted for SOFA score. Tick marks along the x-axis show the individual PLA values of all 78 patients (orange: non-survivors at day 30; green: 30-day survivors) and thus the density of observations underlying each section of the curve. The hazard function is U-shaped, with the lowest estimated risk in the centre of the distribution and increasing risk towards both extremes (p for non-linearity = 0.015). Confidence intervals widen at both ends of the distribution, where observations are sparse

Following successful in vitro validation of the PLA assay, NLRP3 inflammasome activity was quantified in PBMCs obtained from 78 septic patients (Additional File 1). The median age of the total cohort was 64 years (IQR 56–78), and 63% were male. Disease severity at ICU admission was moderate to severe, with a median SOFA score of 8 (IQR: 4–12) and a median SAPS II score of 38 (IQR: 28.5–45). The median ICU length of stay was 12 days (IQR: 5–24). Based on PLA signal intensity, patients were stratified into three groups reflecting low (n = 28), intermediate (n = 23), and high (n = 27) NLRP3 inflammasome activity (Fig. 1). Thus, the PLA signal distribution constitutes the defining variable for group allocation. To transparently illustrate the internal distribution of the stratifying biomarker, PLA signals per cell were descriptively analysed within each data-derived stratum (Fig. 2). Consistent results were obtained when PLA activity was analysed as a continuous variable using restricted cubic splines, demonstrating that the observed U-shaped relationship was not dependent on the chosen categorization (Fig. 3).

Baseline demographic characteristics did not differ significantly between groups. Median age ranged from 63 to 66 years (p = 0.246), and the proportion of male patients was comparable across categories (61–67%, p = 0.877). Similarly, clinical severity scores at admission, including SOFA and SAPS II, were evenly distributed among the three groups (SOFA p = 0.499; SAPS II p = 0.282). ICU length of stay was also comparable (p = 0.844). Inflammatory and laboratory parameters at admission were elevated across the cohort but did not differ significantly between NLRP3 activity groups (Table 1). Relative lymphocyte and monocyte counts did not differ significantly across the three inflammasome activity strata (p = 0.148 and p = 0.173, respectively), indicating that the cellular composition of the analysed PBMC fraction was comparable between groups. Although IL-18 concentrations tended to be higher in the high-activity group, this difference did not reach statistical significance (p = 0.107). PLA-derived inflammasome activity showed a weak but statistically significant correlation with circulating IL-18 (Spearman ρ = 0.342, p = 0.003) and no correlation with IL-1β (ρ = 0.093, p = 0.422; n = 78 for both). Neither cytokine differed significantly between the three activity strata (Table 1), indicating that the soluble markers do not reproduce the stratification obtained by direct quantification of NLRP3–caspase-1 complex assembly. Regarding comorbidities, prevalence rates were similar between groups. Notably, obesity was more frequent in the high NLRP3 activity group (52%) compared with the intermediate (26%) and low (21%) groups (p = 0.046). Consistently, body mass index was significantly higher in the high-activity group (median 30.3 kg/m^2^, IQR 27.3–33.7) than in the intermediate (27.1 kg/m^2^, IQR 24.8–29.7) and low-activity groups (26.6 kg/m^2^, IQR 24.1–29.4; p = 0.036). The primary source of infection was pulmonary in most patients (65%), followed by intra-abdominal infections (14%). The distribution of infection foci did not differ significantly between NLRP3 activity groups. A comprehensive overview of baseline characteristics stratified by NLRP3 activity category is provided in Table 1.

**Table 1:** Baseline characteristics

**Variable**	**Total Cohort (n = 78)**	**Low NLRP3 (n = 28)**	**Intermediate NLRP3 (n = 23)**	**High NLRP3 (n = 27)**	**p-value**
Demographic Data					
Male sex, n (%)	49 (63)	17 (61)	14 (61)	18 (67)	0.877
Age, median (IQR)	64 (56-78)	65 (59–84)	66 (58–78)	63 (52–72)	0.246
Body mass index [kg/m^2^], median (IQR)	28.0 (25.1-31.5)	26.6 (24.1-29.4)	27.1 (24.8-29.7)	30.3 (27.3-33.7)	0.036
**Clinical Parameters, at baseline**					
SOFA score, median (IQR)	8 (4-12)	7.5 (5–12)	7(4–10)	9 (4–12)	0.499
SAPS2 score, median (IQR)	38 (28.5–45)	38 (26.5–45)	35 (26.5–41)	39 (33–47)	0.282
ICU stay, median (IQR)	12 (5-24)	15 (6–20)	11 (4–29)	12 (6–27)	0.844
Septic Shock, n (%)	20 (26)	7 (25)	4 (17)	9 (33)	0.435
Vasopressor requirement, n (%)	52 (67)	18 (64)	15 (65)	19 (70)	0.878
Mechanical ventilation , n (%)	36 (46)	12 (43)	9 (39)	15 (56)	0.463
**Laboratory results, on admission**					
CRP [mg/dL], median (IQR)	14 (11-25)	21 (14–34)	12 (9–20)	14 (11–25)	0.277
PCT [ng/mL], median (IQR)	0.8 (0.4–13.3)	2.4 (0.9–10.7)	0.8 (0.4–13.3)	3.3 (1.1–7.4)	0.712
Lactate [mmol/L], median (IQR)	1.3 (0.9–1.8)	1.5 (0.9–2.1)	0.9 (0.8–1.9)	1.6 (1.2–1.8)	0.636
Creatinine (mg/dL), median (IQR)	1.9 (0.9–3.2)	1.9 (0.7–2.8)	1.1 (0.9–2,0)	2.4 (1.1–5.2)	0.293
Total bilirubin (mg/dL), median (IQR)	0.5 (0.4–1.6)	0.5 (0.4–1.5)	0.7 (0.4–1.9)	0.5 (0.4–1.8)	0.889
Leukocyte count [10^9^/l], median (IQR)	14 (11–18)	13 (11–16)	14 (8–16)	17 (9–19)	0.433
Lymphocytes % of Leukocytes, median (IQR)	9.3 (6.1-13.5)	9.5 (5.5-13.6)	11.1 (7.2-14.9)	8.6 (6.3-11.5)	0.148
Monocytes % of Leukocytes, median (IQR)	5.9 (4.7-7.3)	6.4 (5.3-7.5)	5.8 (5.3-8.2)	5.5 (4.0-6.9)	0.173
IL-6 [pg/mL], median (IQR)	144 (43–431)	225 (102–468)	147 (25–395)	114 (43–338)	0.519
IL-10 [pg/mL], median (IQR)	5.9 (0–17.3)	9.0 (1.1–16.5)	4.9 (1.7–18.3)	6.5 (0.1–17.5)	0.870
IL-1β [pg/mL], median (IQR)	6.0 (0–9.3)	3.7 (0.1–7.6)	6.8 (1.1–10.9)	8.4 (2.4–10.5)	0.186
IL-18 [pg/mL], median (IQR)	210 (113–603)	161 (77–315)	184 (94–672)	528 (169–1040)	0.107
**Comorbidities (%)**					
Hypertension	56 (72)	18 (64)	17 (74)	21 (78)	0.649
Cardiovascular	27 (35)	11 (39)	6 (26)	10 (37)	0.537
COPD	9 (12)	2 (7)	3 (13)	4 (15)	0.678
Chronic kidney disease	13 (17)	2 (7)	5 (22)	6 (22)	0.264
Diabetes	23 (29)	5 (18)	8 (35)	10 (37)	0.274
Alcoholism	4 (5)	0	2 (9)	2 (7)	0.313
Malignancy	16 (21)	4 (14)	5 (22)	7 (26)	0.597
Obesity	26 (33)	6 (21)	6 (26)	14 (52)	0.046
**Focus of Infection (%)**					
Pulmonary	52 (67)	16 (57)	15 (65)	21 (78)	0.224
Intra-abdominal	11 (14)	6 (21)	4 (18)	1 (4)	
Skin and soft tissue	4 (6)	0	1 (4)	3 (11)	
Urogenital	2 (3)	2 (7)	0	0	
Central nervous system	2 (3)	1 (4)	1 (4)	0	
Other / Unknown	7 (9)	3 (11)	2 (9)	2 (7)	

Median NLRP3–Caspase-1 interactions per cell increased stepwise across groups with 0.270 (IQR 0.169–0.401) signals per cell in the low group, 0.673 (IQR 0.603–0.757) in the intermediate group, and 1.565 (IQR 1.231–1.977) in the high group (p<0.001, Fig. 2). These data primarily serve to visualize the distribution characteristics of the underlying stratification variable and to demonstrate the internal consistency of the inflammasome activity categorization.

**Fig. 4:**
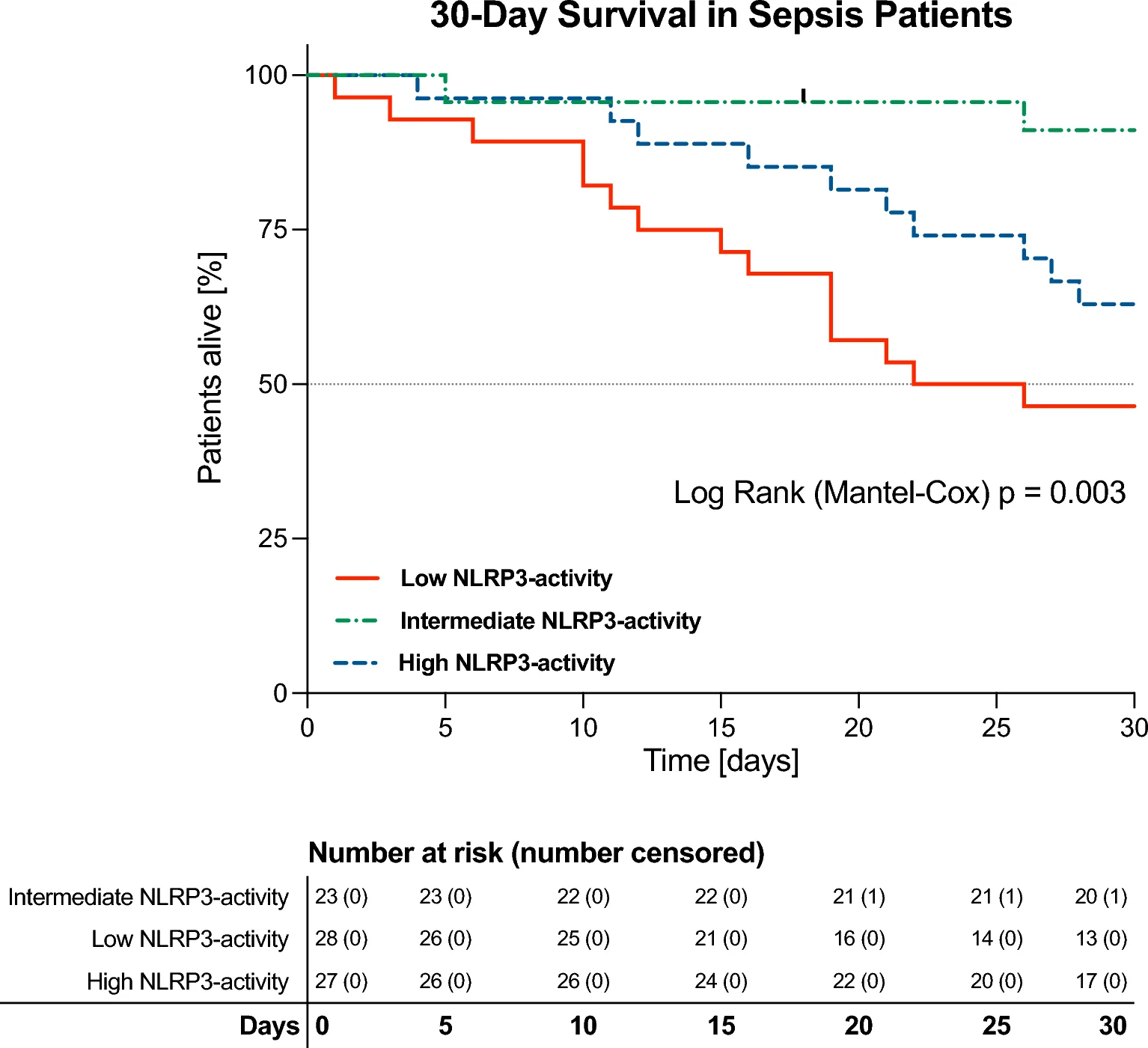
Kaplan-Meier survival according to NLRP3 inflammasome activity comparing between the three inflammasome activation strata. 30-day survival in septic patients with low (red), intermediate (green) or high (blue) NLRP3 inflammasome activity; between-group comparison by log-rank test. Numbers at risk are given below the x-axis. Within 30 days, 15 of 28 patients (54%) in the low-activity, 2 of 23 (9%) in the intermediate-activity and 10 of 27 (37%) in the high-activity stratum died, corresponding to 27 deaths in the total cohort. One further patient in the intermediate-activity stratum was censored before day 30 (tick mark on the survival curve); three patients therefore left the risk set in this stratum, of whom two died. No censoring before day 30 occurred in the low- or high-activity strata

Kaplan–Meier survival analysis demonstrated differences in 30-day survival across the three groups, with the most favourable outcomes observed in patients with intermediate NLRP3 activity. Both low (≤0.51 PLA signals per cell) and high (≥1.00 signals per cell) inflammasome activation was associated with increased mortality compared with the intermediate group (>0.51 to <1.00 signals per cell), consistent with a non-linear (log-rank p < 0.05, Fig. 4) or even U-shaped risk distribution (Fig. 3).

Cox proportional hazards regression was used to compare 30-day mortality across the three data-derived tertiles of inflammasome activity based on NLRP3 activation. The proportional hazards assumption was assessed using Schoenfeld residuals and showed no evidence of violation for any covariate (all p > 0.05). After adjustment for relevant clinical covariates, both low inflammasome activity (HR 7.88, 95% CI 1.79–34.65; p = 0.006) and high inflammasome activity (HR 4.35, 95% CI 1.01–19.88; p = 0.048) remained associated with increased 30-day mortality (Table 2). Within 30 days, 27 of 78 patients died (35%), including 15/28 (54%) in the low-, 2/23 (9%) in the intermediate-, and 10/27 (37%) in the high-activity group. Given the limited number of events and data-driven model selection, estimates were unstable with wide confidence intervals and should be considered hypothesis-generating.

**Table 2:** Cox regression analysis in sepsis patients (n=78)

**Covariable**	**Single predictor**		**Multiple predictors**			
			**Initial**		**Restricted**	
	**Hazard Ratio (95% CI)**	**p-value**	**Hazard Ratio (95% CI)**	**p-value**	**Hazard Ratio (95% CI)**	**p-value**
NLRP3 inflammasome activity Intermediate Low High	1 8.37 (1.92-36.8) 4.66 (1.02-21.3)	- 0.005 0.047	1 7.48 (1.66-33.7) 4.94 (1.04-23.5)	- 0.009 0.045	1 7.88 (1.79-34.65) 4.35 (1.01-19.88)	- 0.006 0.048
Sex Women Men	1 1.74 (0.82-3.69)	- 0.153	1 1.57 (0.69-3.59)	- 0.284		
Age [years]	1.02 (0.99-1.05)	0.168	1.01 (0.98-1.04)	0.514		
Obesity (BMI ≥ 30 kg/m²) No Yes	1 0.95 (0.42-2.13)	0.899	1 1.39 (0.61-3.16)	- 0.432		
SOFA score [per unit]	1.17 (1.06-1.28)	0.002	1.16 (1.05-1.28)	0.003	1.15 (1.05-1.27)	0.003

Hazard ratio point estimates, 95% CI, and p-values (two-sided) are reported, SOFA: Sequential Organ Failure Assessment. The model significantly improved model fit compared to the null model (likelihood ratio χ^2^ = 22.0, p < 0.001)

To confirm that the U-shaped association was not an artefact of tertile-based categorisation, PLA activity was additionally modelled as a continuous variable using restricted cubic splines (Fig. 3). The estimated hazard function was distinctly non-linear (p for non-linearity = 0.015), with the lowest mortality risk in the central part of the PLA distribution and increasing hazard ratios towards both the lower and the upper end of the observed range. The nadir of the curve was located at approximately 1.2 signals per cell, close to the boundary between the intermediate and high strata. The spline model therefore reproduced the U-shaped risk pattern of the categorical analysis without relying on any pre-specified cut-points (Fig. 3).

## Discussion

In this multicentre proof-of-concept pilot study of patients with sepsis, we found that NLRP3 inflammasome activity in peripheral blood mononuclear cells was associated with 30-day survival in a non-linear manner. Patients with intermediate inflammasome activity had the most favourable outcome, whereas both low and high inflammasome activity were associated with increased mortality, even after adjustment for relevant clinical covariates including SOFA score. These data support the concept that inflammasome signalling in sepsis is not simply “good” or “bad,” but instead follows a biologically plausible double-edged or even U-shaped pattern: too little inflammasome activity may reflect insufficient innate immune competence, whereas too much activity may drive collateral inflammatory tissue injury. This observation is important because it directly links a functional, cell-specific readout of innate immune activation to outcome and reinforces the broader concept that sepsis is not a uniform immunological entity, but a heterogeneous and dynamic syndrome composed of divergent host-response trajectories. Contemporary sepsis frameworks increasingly emphasize this heterogeneity and the need for informative subtyping to guide immunomodulatory interventions [3].

A major strength of our study is that it moves beyond conventional soluble biomarkers and assesses inflammasome activation at the level of functional complex assembly in individual cells. This distinction matters. Much of the existing clinical literature has relied on circulating cytokines such as IL-1β or IL-18 as surrogate readouts of inflammasome activity. However, these mediators are influenced by release kinetics, receptor binding, systemic dilution, organ clearance, and inflammasome-independent sources, which limits their precision as markers of cell-specific inflammasome function. Even ASC-speck detection, although closer to the cellular event, remains less specific than direct visualization of NLRP3–Caspase-1 proximity and may capture incomplete or noncanonical inflammasome-related structures [14]. By contrast, PLA enables spatially resolved detection of inflammasome assembly within intact PBMCs and therefore provides a more proximal and mechanistically meaningful measure of functional NLRP3 activation. This is especially relevant in sepsis, where overlapping danger signals and pathogen-derived stimuli activate multiple innate pathways simultaneously and where conventional plasma biomarkers often fail to capture the true complexity of the host response [15].

The association between high inflammasome activity and poor outcome is biologically intuitive and consistent with a large body of experimental work [16–20]. NLRP3 activation leads to caspase-1 cleavage, maturation of IL-1β and IL-18, and gasdermin D-mediated pyroptosis, thereby amplifying cytokine release, endothelial activation, immunothrombosis, mitochondrial dysfunction, and tissue injury [21, 22]. In experimental sepsis, excessive NLRP3 signalling has been linked to acute lung injury, septic cardiomyopathy, acute kidney injury, and endothelial barrier failure [22]. Mechanistically, exaggerated inflammasome activation can generate a feed-forward loop in which DAMP release, mitochondrial damage, and oxidative stress perpetuate further NLRP3 activation, thus promoting organ dysfunction beyond what is required for pathogen control [12]. Our finding that patients with high NLRP3 activity had lower survival rates than those with intermediate activation fits well with this concept of maladaptive hyperinflammation and collateral host injury. Notably, recent reviews likewise emphasize that moderate NLRP3 activation may support pathogen clearance, whereas excessive activation contributes to cytokine storm, organ failure, and death during the acute phase of sepsis [22]. An additional observation that merits consideration is that obesity was markedly more frequent in the high-activity stratum, accompanied by a correspondingly higher body mass index (Table 1). Although these findings do not establish causality, they are biologically plausible given the well-described link between excess adiposity and chronic priming of the NLRP3 inflammasome [23]. Adipose tissue provides persistent metabolic danger signals, including saturated fatty acids, ceramides, cholesterol crystals, and hyperglycaemia, while adipose tissue macrophages may sustain low-grade inflammasome activity [24]. Part of the high-NLRP3 phenotype may therefore reflect a pre-existing metabolic-inflammatory state that precedes and potentially modulates the acute host response to infection, rather than representing the intensity of the acute infectious stimulus alone. Importantly, obesity itself was not associated with 30-day mortality in either univariable or multivariable analysis, and the association between inflammasome activity and outcome persisted after adjustment (Table 2), arguing against obesity acting as a simple confounder of the observed U-shaped relationship. This distinction may nevertheless be clinically relevant in the context of inflammasome-directed therapies, as patients with a pre-existing metabolic-inflammatory priming state may differ biologically, and potentially therapeutically.

At the same time, the more intriguing and perhaps clinically even provocative finding of our study is that low inflammasome activity was also associated with markedly increased mortality. This observation argues strongly against a simplistic interpretation in which inflammasome activation is viewed only as a pathogenic driver. Instead, our data support the idea that insufficient NLRP3 activation at sepsis onset may reflect a state of impaired innate immune competence. Sepsis is now understood as a dynamic immune disorder in which hyperinflammation, and immunosuppression can coexist from very early time points rather than occurring as neatly separated sequential phases [25]. Reduced monocyte HLA-DR expression, impaired cytokine production, defective antigen presentation, lymphocyte dysfunction, and susceptibility to secondary infection all characterize sepsis-associated immunoparalysis [26]. In this context, diminished NLRP3 assembly in circulating immune cells may be a functional correlate of an exhausted or reprogrammed innate immune compartment that is no longer able to mount an effective inflammatory response when needed. This interpretation aligns with broader conceptual models of sepsis-induced immune dysfunction and with current translational efforts to identify immune endotypes rather than treat sepsis as one monolithic inflammatory disease [27]. This interpretation, however, remains hypothetical, as total leukocyte counts and IL-10 concentrations were the only parameters available in our dataset that could be related to impaired innate immune competence, and neither differed across the activity groups (Table 1). Notably, even the clinical study most closely related to ours did not establish such a causal link [11]. In that study, reduced basal and nigericin-induced ASC-speck formation and caspase-1 activation were observed in non-survivors on day 0. Although mHLA-DR and CD4⁺ T-cell counts were assessed in the same cohort, no association with inflammasome activity was reported [11]. The proposition that reduced NLRP3–caspase-1 assembly reflects impaired innate immune competence should therefore be regarded as biologically plausible but remains unproven. Definitive evidence will require studies combining PLA-based profiling with established markers of immunosuppression and clinical endpoints, such as secondary infection and pathogen clearance.

Our data are also concordant with emerging human studies showing that impaired inflammasome responsiveness is associated with adverse outcome. Cui and colleagues reported that lower absolute numbers of ASC-speck-positive monocytes on day 6 after sepsis onset were associated with increased mortality, suggesting that insufficient in vivo inflammasome activation identifies a high-risk subgroup [14] . Likewise, Coudereau et al. found that diminished basal expression and ex vivo activation of NLRP3 in circulating myeloid cells at day 0 were linked to higher 28-day mortality in bacterial and viral sepsis [11]. Garnacho-Montero et al. further observed that higher NLRP3 protein levels in PBMCs later during the course of sepsis were associated with improved survival [28]. Taken together, these studies, despite differences in timing and methodology, converge on the idea that low inflammasome activity can be harmful and may signify immune failure rather than protection. Our study extends these observations by providing a direct in situ measure of NLRP3–Caspase-1 assembly at sepsis onset and by integrating both ends of the distribution into one clinically coherent model: mortality risk appears to rise at both extremes, with an intermediate zone of activation associated with the best outcome.

The observation that NLRP3 inflammasome activity was associated with mortality after adjustment for SOFA score carries direct translational significance. SOFA quantifies the downstream consequences of immune dysregulation, namely organ dysfunction severity, hemodynamic compromise, and respiratory failure, but does not capture the upstream biological state that drives these consequences. The strong impact on outcome of the PLA-derived inflammasome activity suggests that functional immune profiling provides a mechanistically distinct dimension of patient characterisation. This distinction matters clinically because the two high-risk subgroups identified in this study, patients with excessive inflammasome activation and those with insufficient activation, represent diametrically opposed immune states likely requiring opposite therapeutic interventions. The broader precision medicine literature increasingly recognises that treatment failure in previous sepsis immunomodulation trials may reflect the absence of biologically informed patient selection rather than a failure of the underlying therapeutic rationale [29, 30]. In this context, the ImmunoSep trial has demonstrated that aligning immunomodulatory therapy with the patient’s dominant immune phenotype can improve outcomes [31]. PLA-based inflammasome profiling could operationalise precisely this principle: patients with high NLRP3 activity represent a biologically coherent target population for pharmacological inflammasome inhibition, supported by a substantial body of preclinical evidence demonstrating that selective NLRP3 blockade attenuates cytokine release, organ injury, and mortality in experimental sepsis [18–20, 32], whereas patients with low inflammasome activity are precisely those in whom such blockade would be contraindicated and immune-stimulating interventions such as IL-7 or granulocyte-macrophage colony-stimulating factor more appropriate.

These considerations are hypothesis-generating rather than practice-guiding. An observational association between inflammasome activity and outcome does not establish that modifying this pathway will improve outcomes. Accordingly, the potential use of NLRP3 activity to guide NLRP3 blockade or immune-enhancing strategies should be regarded as a testable hypothesis requiring prospective validation in adequately powered, predictive-enrichment trials.

As an observational proof-of-concept pilot study, this analysis was designed to establish methodological feasibility and generate hypotheses rather than provide definitive clinical thresholds or effect size estimates. Furthermore, sampling was anchored to sepsis diagnosis rather than disease onset and therefore may not accurately reflect the individual biological onset of disease. Given that inflammasome activity is dynamic and may vary with illness phase, this may complicate the interpretation of our findings. Moreover, PLA does not distinguish between PBMC subsets, which may differ in inflammasome activation. As NLRP3 inflammasome activity is concentrated in monocytes, the PLA signal per cell represents a composite measure across the PBMC fraction rather than a per-monocyte activity, although the relative monocyte and lymphocyte fractions did not differ between strata (Table 1). Two further constraints apply: inflammasome activity in circulating cells need not reflect events in the affected tissues, and complex assembly is a structural event that does not in itself establish downstream functional consequences in vivo. Future adaptations incorporating cell-specific markers or flow cytometry-based PLA may provide greater cellular resolution. Longitudinal sampling may further clarify whether dynamic changes in inflammasome activity correlate with disease progression, treatment response, or recovery. Because no validated clinical thresholds for PLA-derived NLRP3 inflammasome activity currently exist, patients were stratified using data-derived tertiles. The exploratory nature and small sample size limit the precision of the spline estimates, particularly at the extremes of the PLA distribution. The identified activity ranges should therefore not be considered definitive clinical thresholds. Larger independent cohorts with blinded duplicate readings, technical replicates, and assessment of intra- and inter-assay variability are needed to establish robust and reproducible cut-points.

## Conclusions

In conclusion, this proof-of-concept pilot study demonstrates that NLRP3 inflammasome activity in PBMCs at sepsis onset is associated with 30-day mortality in a non-linear manner, with both low and high levels of activity linked to adverse outcomes. These findings establish the translational feasibility of proximity ligation assay-based inflammasome profiling in a clinical sepsis cohort and provide proof-of-concept that direct quantification of NLRP3-caspase-1 complex formation captures biologically relevant immune heterogeneity not resolvable by standard inflammatory biomarkers. Future validation studies in larger, adequately powered independent cohorts should confirm the observed associations. If confirmed, PLA-based inflammasome profiling may provide a mechanistically grounded basis for patient stratification in future immunomodulatory trials. The therapeutic implications of this concept remain hypotheses generated by this observational pilot study and require prospective validation before clinical application.

## Methods

### Study design, study participants and data collection

This prospective, multicentre proof-of-concept pilot study was conducted in ICUs of three participating German university hospitals between June 2019 and April 2020. The study was approved by the Ethics Committee of the Medical Faculty of Ruhr University Bochum (No. 18-6257 and No. 18-6606–BR). Written informed consent was obtained from all participants or legal guardians. This study was conducted in accordance with the Declaration of Helsinki, good clinical practice guidelines, and local regulatory requirements.

Patients with sepsis were considered eligible if they fulfilled the criteria for sepsis as defined by the current Third International Consensus Definitions for Sepsis and Septic Shock definition [1]. In addition, enrolment, written informed consent, and first blood sampling had to be completed within the first 24 h after diagnosis of sepsis. Assuming that the disease severity of sepsis at the time of ICU admission was associated with the extent of inflammasome activity at that time, we assessed the SOFA score on day 1 at the same time as collecting blood. In this context, the SOFA score was calculated as described by Lambden et al [33], using the last Glasgow Coma Scale score before intubation in patients under sedation. In all cases, blood samples were processed directly and PBMCs were isolated, as described in the next section. Exclusion criteria were as follows: < 18 years of age, pregnancy, preexisting anaemia, and decision to withhold or withdraw life-sustaining treatment on the day of study inclusion. A total of 78 patients with sepsis treated in the ICU in one of the participating study centres were enrolled in this study (Additional file 1).

All Patients were followed for 30-day survival commencing from the day of the diagnosis of sepsis.

Routine study data were collected from the electronic health records and imported to a central database. Each respective local study site first checked and confirmed the validity of the documented data. After the local study teams had verified their data, the complete data set was additionally validated by an independent intensive care specialist together with a statistician regarding plausibility. Contradictions

were reported as queries and again rechecked and solved by the local study teams as part of the data clearance process.

### PBMC isolation

PBMCs from 78 randomly selected SepsisDataNet.NRW patients were isolated via Ficoll density gradient centrifugation (GE Healthcare, Germany). After erythrocyte lysis using distilled water, the cells were washed with PBS, and the concentration of live and dead cells was determined using trypan blue staining. An absolute cell count of 5×10^5^ trypan blue-negative/live cells was transferred to microscope slides using a Cytospin centrifuge (Cellspin II, Tharmac, Germany). The slides were fixed in 4% formaldehyde at 4 °C for 30 min, air-dried, and stored at −80 °C until analysis.

### Measurement of cytokines in serum

Serum cytokine levels were measured at recruitment using the LEGENDplex™ Human Inflammation Panel 1 (BioLegend, USA) per manufacturer’s instructions. After incubation with capture beads and detection antibodies, fluorescence was analysed via flow cytometry (BD FACSCanto II, BD Biosciences).

### NLRP3 inflammasome proximity ligation assay

Fixed cells were permeabilized with 0.1% Triton X-100 and 1% SDS (Carl Roth, Germany) to enhance epitope accessibility, followed by blocking with Duolink® Blocking Solution (Sigma-Aldrich, USA). Slides were incubated overnight at 4 °C with primary antibodies directed against NLRP3 (AdipoGen, USA) and Caspase-1 (Cell Signaling Technology, UK), each at a dilution of 1:200. After washing with TBST, species-specific, in-house–conjugated proximity probes (Mouse+/Rabbit−; Jackson ImmunoResearch, UK) were applied at 32 °C. Hybridization and ligation of S3 oligonucleotides (Biomers.net, Germany), followed by rolling circle amplification using Phi29 polymerase (Sigma-Aldrich, Germany), were performed at 32 °C. Detection was achieved using S3-DO Green (Biomers.net, Germany), DAPI, and Phalloidin (Invitrogen™, Germany).

As this study represents the first application of a proximity ligation assay (PLA) to quantify NLRP3 inflammasome assembly in human samples, we additionally performed a rigorous in vitro validation prior to its implementation in septic patients (Additional file 2). Specifically, we established and validated the PLA in THP-1-derived macrophages to ensure that the detected NLRP3–Caspase-1 proximity signals accurately reflect functional inflammasome activation. Canonical inflammasome activation was induced by priming cells with lipopolysaccharide (LPS), followed by stimulation with ATP or nigericin. PLA-derived signals were systematically compared with established reference methods for inflammasome activity assessment, including caspase-1 activity assays, multiplex cytokine quantification of IL-1β and IL-18, and immunoblot detection of cleaved Caspase-1 and Gasdermin D. Concordance with these established readouts confirmed that the PLA reliably reflects canonical NLRP3 inflammasome activation and provided the methodological foundation for its subsequent application in clinical samples. Detailed experimental protocols and validation results are provided in Additional file 2.

### Image analysis

Fluorescence images were acquired using an inverted microscope (IX51, Olympus) as five-plane z-stacks. Maximum intensity projections were analysed with FIJI and a custom CellProfiler pipeline. Phalloidin (TRITC) was utilised to identify F-actin–containing cytoskeletal structures, and DAPI staining was subsequently employed to identify nucleus-containing cells within these regions. PLA signals (FITC) were quantified per cell using a predefined automated pipeline. Green‑channel (FITC) signals were enhanced as 10‑pixel speckles, and PLA‑positive objects (2–15 pixels) were identified by global Minimum Cross‑Entropy thresholding (intensity range 0.15–1.0). Objects outside this size range or touching the image border were excluded. The PLA channel exposure time and gain were kept constant (1 second; fixed gain) for all samples. The median of 182 cells per patient was analysed, which exceeds the originally stated minimum of 50 cells per sample. Image acquisition and quantitative analysis were performed blinded to clinical outcome; the investigator was unaware of 30-day survival status, and outcome data were linked to the dataset only after completion of all image analyses.

### Statistical analyses

Continuous variables are presented as median with interquartile range (IQR) and categorical variables as absolute numbers and percentages. Comparisons across the three NLRP3 inflammasome activity strata were performed using the Kruskal–Wallis test for continuous variables and the χ^2^ test or, where expected cell frequencies were < 5, Fisher’s exact test (Freeman–Halton extension) for categorical variables. Correlations between PLA-derived inflammasome activity and circulating cytokine concentrations (IL-1β, IL-18) were assessed using Spearman’s rank correlation coefficient (ρ). As the distribution of PLA signals per cell across strata is determined by the stratification itself, this comparison (Fig. 2) is presented descriptively.

As this proof-of-concept pilot study was designed to establish methodological feasibility and generate hypotheses, no a priori power calculation was performed. Because no established clinical thresholds for PLA-derived NLRP3 inflammasome activity exist, patients were stratified into tertiles based on the empirical distribution of PLA signals within the study cohort. This exploratory categorization was chosen to facilitate assessment of potential non-linear associations between inflammasome activity and clinical outcome. To evaluate whether the observed association depended on the selected cut-points, PLA activity was additionally modelled as a continuous variable in a Cox proportional hazards model using restricted cubic splines. Three knots were placed at the 10th, 50th and 90th percentiles of the PLA distribution, and the median PLA value served as the reference. The spline model was adjusted for SOFA score, in accordance with the restricted multivariable model shown in Table 2. Non-linearity was assessed by a likelihood-ratio test comparing the full spline model with a model containing the linear term only; the overall association between PLA activity and 30-day mortality was tested by a likelihood-ratio test against the corresponding model without the PLA terms. Hazard ratios with 95% confidence intervals are displayed across the entire observed range of PLA values (Fig. 3).

Survival analyses were conducted using the Kaplan–Meier method, and differences between groups were assessed with the log-rank test. To evaluate the association between clinical variables (including NLRP3 inflammasome activity) and survival, Cox proportional hazards regression analyses were performed using a sequential modelling approach. Candidate variables were selected based on two predefined criteria: (i) variables showing significant differences across the three study groups at baseline, and (ii) variables described in the literature as established or clinically relevant risk factors for mortality in septic patients. This approach ensured both data-driven and clinically informed variable selection. First, univariable Cox regression models were calculated for each predictor separately. Subsequently, a multivariable Cox regression model including all candidate predictors was fitted to assess their joint effects on survival. To reduce the risk of overfitting, a restricted final model was then constructed including only those variables that demonstrated a statistically significant association with survival (p ≤ 0.05) in either the univariable or the initial multivariable analysis. Hazard ratios (HRs) with corresponding 95% confidence intervals (CIs) are reported. No adjustment for multiplicity was performed. All p-values are two-sided and considered statistically significant at an a priori alpha level of 0.05. The proportional hazards assumption was assessed using Schoenfeld residuals and was not violated. All analyses were performed using SPSS, Version 31.0. AI-assisted tools were used solely for spell-checking and linguistic refinement; no AI was used for data analysis or interpretation.

## Supplementary Information

Additional file 1: Flow diagram illustrating patient screening, eligibility assessment, study inclusion, and allocation into NLRP3 inflammasome activity groups. A total of 121 patients were screened, of whom 80 met the inclusion criteria and 78 were included in the final analysis. Patients were subsequently stratified according to NLRP3 inflammasome activity into low (n = 28), intermediate (n = 23), and high activity groups (n = 27).

Additional file 2: In-vitro validation of the NLRP3–Caspase-1 proximity ligation assay. Experimental validation of the NLRP3–Caspase-1 PLA using THP-1-derived macrophages stimulated with canonical NLRP3 activators. Inflammasome activation was induced by LPS priming followed by ATP or Nigericin stimulation. PLA signal intensity was compared with established readouts of inflammasome activation, including caspase-1 activity, IL-1β and IL-18 release, and protein cleavage of caspase-1 and gasdermin D, demonstrating concordant activation patterns across all assays.

## Data Availability

The datasets generated and/or analysed during the current study are not publicly available due to patient privacy and ethical restrictions but are available from the corresponding author on reasonable request.

## Abbreviations

ASC: Apoptosis-associated speck-like protein containing a caspase recruitment domain
ATP: Adenosine triphosphate
BMI: Body mass index
CI: Confidence interval
COPD: Chronic obstructive pulmonary disease
CRP: C-reactive protein
DAMP: Damage-associated molecular pattern
DAPI: 4′,6-diamidino-2-phenylindole
FITC: Fluorescein isothiocyanate
HR: Hazard ratio
ICU: Intensive care unit
IL: Interleukin
IQR: Interquartile range
LPS: Lipopolysaccharide
NLRP3: NOD-like receptor family pyrin domain containing 3
PBMCs: Peripheral blood mononuclear cells
PBS: Phosphate-buffered saline
PCT: Procalcitonin
PLA: Proximity ligation assay
SD: Standard deviation
SAPS II: Simplified Acute Physiology Score II
SDS: Sodium dodecyl sulfate
SOFA score: Sequential Organ Failure Assessment score
TBST: Tris-buffered saline with Tween 20
THP-1: Human monocytic leukemia cell line THP-1
TRITC: Tetramethylrhodamine isothiocyanate
