# Supplementary material for: Quantification of NLRP3–caspase-1 complex assembly reveals a non-linear association with 30-day mortality in sepsis: a proof-of-concept pilot study
Source: Intensive Care Med Exp. 2026 Oct 29;14:143. doi: 10.1186/s40635-026-00992-4 (PMC13719975; doi:10.1186/s40635-026-00992-4)
Supplement: Supplementary file 1 [file 40635_2026_992_MOESM1_ESM.pdf]

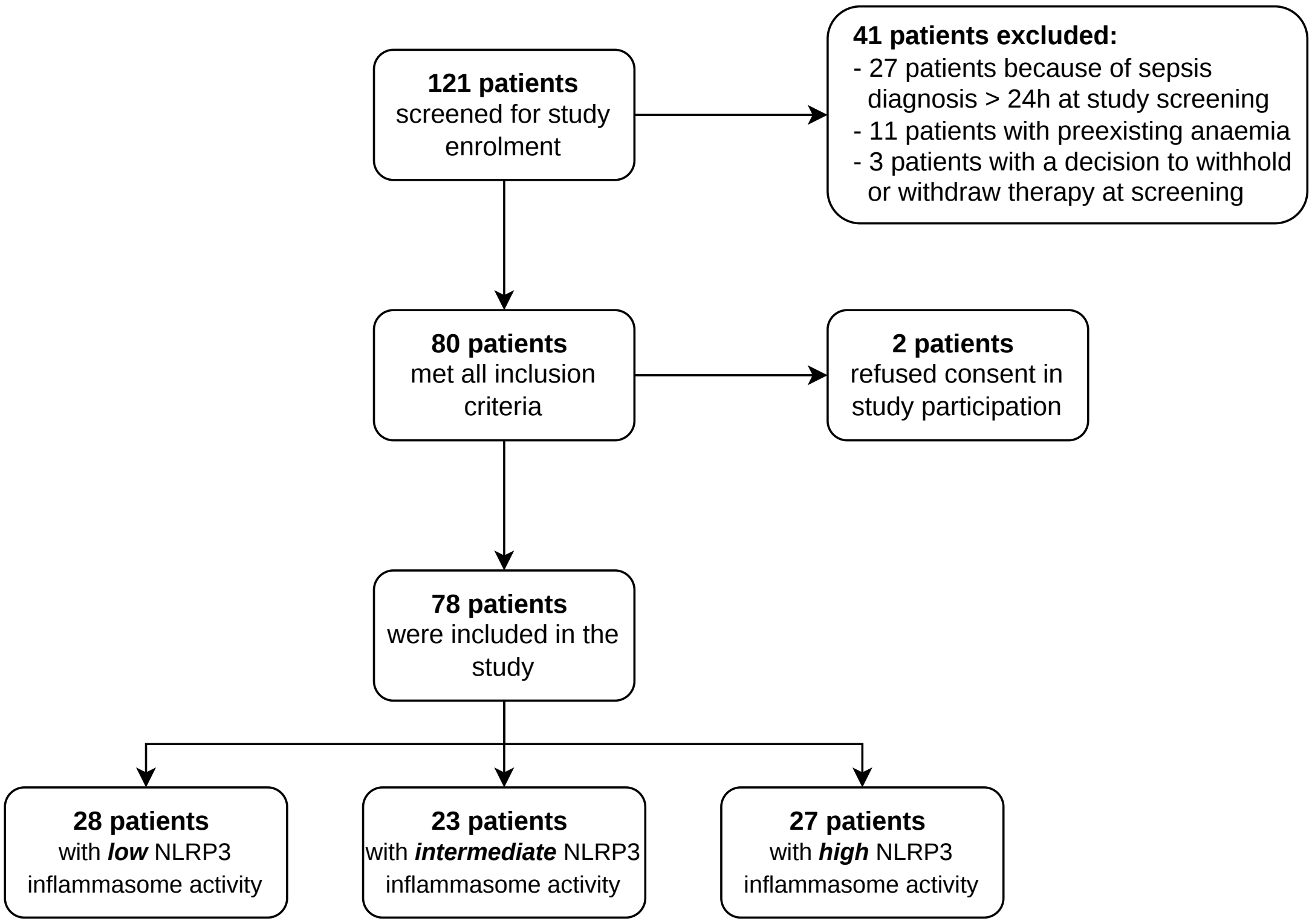
121 patients
screened for study enrolment
41 patients excluded:
- 27 patients because of sepsis diagnosis > 24h at study screening
- 11 patients with preexisting anaemia
- 3 patients with a decision to withhold or withdraw therapy at screening
80 patients
met all inclusion criteria
2 patients
refused consent in study participation
78 patients
were included in the study
28 patients
with ***low*** NLRP3 inflammasome activity
23 patients
with ***intermediate*** NLRP3 inflammasome activity
27 patients
with ***high*** NLRP3 inflammasome activity
