# Supplementary material for: Quantification of NLRP3–caspase-1 complex assembly reveals a non-linear association with 30-day mortality in sepsis: a proof-of-concept pilot study
Source: Intensive Care Med Exp. 2026 Oct 29;14:143. doi: 10.1186/s40635-026-00992-4 (PMC13719975; doi:10.1186/s40635-026-00992-4)
Supplement: Supplementary file 2 [file 40635_2026_992_MOESM2_ESM.pdf]

Supplementary Material

In-vitro validation of the NLRP3-Caspase-1 Proximity Ligation Assay

To establish and validate the Proximity Ligation Assay (PLA) for detection of NLRP3 inflammasome activation, we used a model of THP-1-derived macrophages. Cells were differentiated with PMA and primed with lipopolysaccharide (LPS), followed by secondary stimulation with either ATP or Nigericin to activate the NLRP3 inflammasome. Caspase-1 activity, measured via chemiluminescence, showed no significant increase following LPS priming alone but rose substantially with ATP stimulation and even more strongly with Nigericin. Similarly, IL-1β and IL-18 concentrations in the culture supernatant remained low under basal and LPS-only conditions but increased markedly upon secondary stimulation. Western blot analysis confirmed increased levels of cleaved Caspase-1 (p20 subunit) and cleaved Gasdermin D after ATP or Nigericin treatment, but not with LPS alone. The PLA was then applied to the same conditions to visualize NLRP3–Caspase-1 complex formation at the single-cell level. As expected, a minimal non significant PLA signal was observed in unstimulated or LPS-only treated cells. In contrast, cells stimulated with LPS + ATP or LPS + Nigericin showed a robust increase in PLA signal, indicating formation of the NLRP3–Caspase-1 complex. Quantitative image analysis revealed strong and statistically significant correlations between PLA signal intensity and other readouts of inflammasome activation. Specifically, PLA signal correlated with Caspase-1 activity ($r_s = 0.689$, $p < 0.001$), IL-1β ($r_s = 0.693$, $p < 0.001$), and IL-18 ($r_s = 0.667$, $p < 0.001$). On the protein level, cleaved Caspase-1 correlated very strongly with PLA intensity ($r_s = 0.815$, $p < 0.001$), and cleaved Gasdermin D showed a moderate but significant correlation ($r_s = 0.484$, $p < 0.001$). Together, these data demonstrate that the PLA specifically detects canonical NLRP3 inflammasome activation in vitro. Its strong correlation with both functional and molecular markers supports its use as a sensitive and specific assay for inflammasome activity. Based on these findings, the assay was subsequently applied to primary PBMC samples from septic patients.

S.1 Cell Culture

The human monocytic cell line THP-1 was used as an in vitro model for PBMCs. Cells were cultured continuously in RPMI-1640 medium (PAN-Biotech Aidenbach, Germany) supplemented with 10% fetal calf serum (FCS) (PAN-Biotech Aidenbach, Germany) and 1% penicillin/streptomycin (Thermo Fisher Scientific, Bremerhaven, Germany) at 37 °C in a humidified atmosphere containing 5% CO_2. To induce differentiation into macrophage-like cells, THP-1 cells were treated with 50 ng/mL phorbol 12-myristate 13-acetate (PMA) (Sigma-Aldrich Chemie GmbH, Taufkirchen, Germany) for 24 hours. Following PMA treatment, cells were allowed to rest for an additional 24 hours in standard RPMI-1640 medium containing 10% FCS and 1% penicillin/streptomycin. For inflammasome priming, cells were stimulated with 1 µg/mL lipopolysaccharide (LPS) from Escherichia coli (serotype O55:B5) (Sigma-Aldrich Chemie GmbH, Taufkirchen, Germany) for 3 hours. Subsequently, the NLRP3 inflammasome was activated by treating the cells with 5 mM ATP (Sigma-Aldrich Chemie GmbH, Taufkirchen, Germany) or 10 µM Nigericin from Streptomyces hygroscopicus (Sigma-Aldrich Chemie GmbH, Taufkirchen, Germany) for 1 hour.

S.2 Caspase-1 Assay

Caspase-1 activity was measured using the Caspase-Glo® 1 Inflammasome Assay (Promega, Madison, USA). Cells were seeded into white NUNC 96-well plates and subsequently differentiated and stimulated as previously described. Reagents were prepared according to the manufacturer's instructions, and cells were equilibrated to room temperature for 10 minutes prior to assay setup. Cells were incubated with the Z-WEHD substrate and MG-132 inhibitor to prevent non-specific proteasomal activity. In parallel, half of the wells were additionally treated with the specific Caspase-1 inhibitor Ac-YVAD-CHO to distinguish Caspase-1-dependent luminescence. After 60 minutes of incubation in the dark at room temperature, luminescence was measured at the ELISA-Plate-Reader ClarioStar Plus BMG (Labtech, Ortenberg, Germany) in relative light units (RLU). Caspase-1-specific activity was calculated by subtracting the luminescence of inhibitor-treated wells from that of the corresponding untreated wells. All conditions were tested in technical duplicates, with empty wells serving as background controls. The experiment was repeated independently five times. Results are depicted in Figure S1.

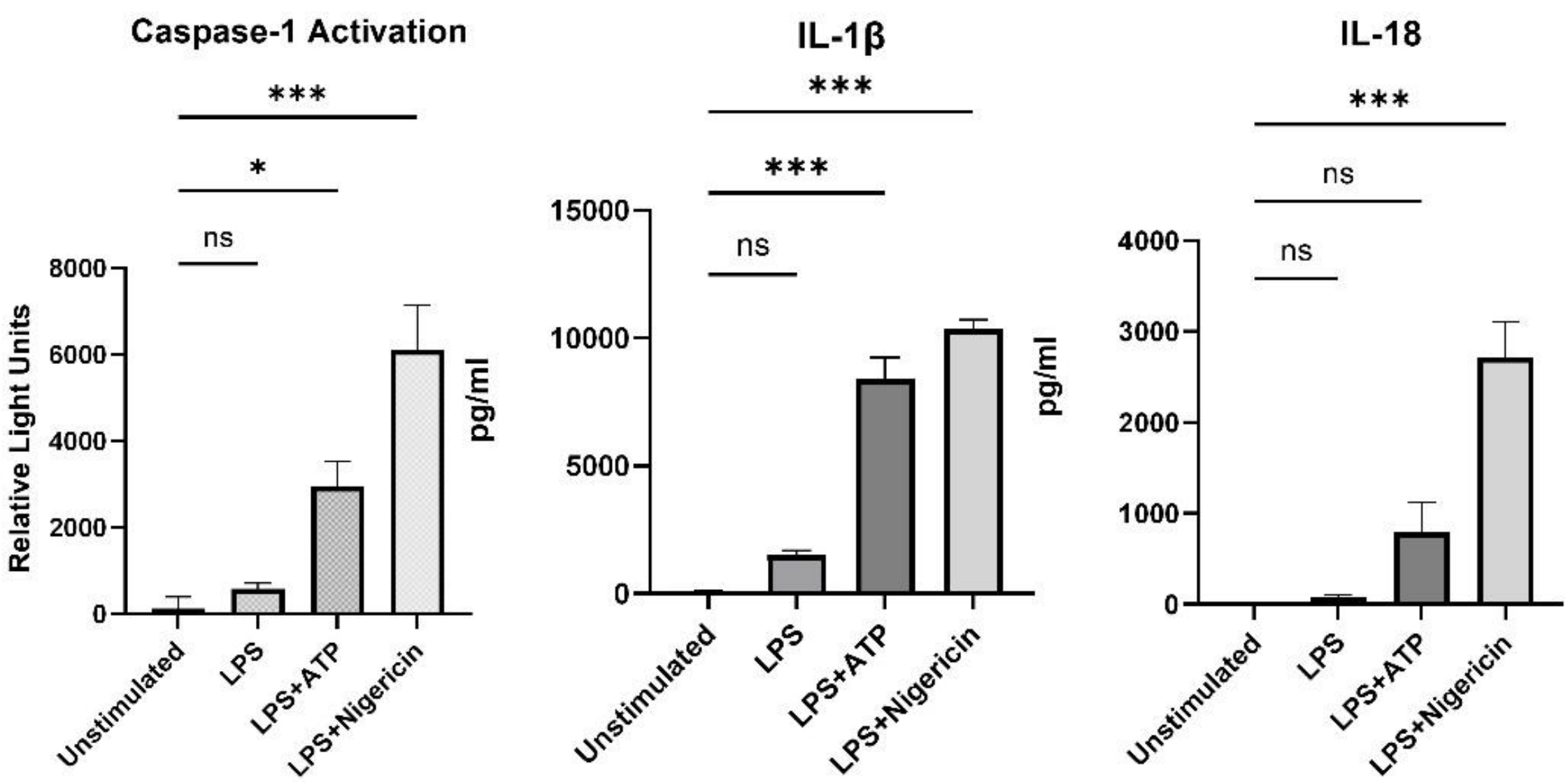

Figure S1: Inflammasome Activation in Stimulated THP-1 Macrophages ***A:*** Chemiluminescence-Assay assessing Caspase-1 activation, ***B:*** Secreted IL-1ß, ***C:*** Secreted IL-18; Mean + SEM, Ordinary One-way ANOVA + Dunn´s Test, n=5, , ns = non-significant, (*) = p < 0,05, (***) = p < 0,001

S.3 Measurement of Cytokines in Cell Culture

After differentiation and stimulation as described above the Medium was collected and centrifugated to remove cell damage. Cytokine levels were measured using the LEGENDplex™ Human Inflammation Panel 1 (BioLegend, San Diego, CA, USA), following the manufacturer's instructions. Briefly, serum samples were incubated with LEGENDplex beads for antigen capture, followed by washing and incubation with detection antibodies. Fluorescence was subsequently measured using a flow cytometer (BD FACSCanto II, BD Biosciences, San Jose, CA, USA). Cytokine concentrations falling below the lower limit of

detection (LOD) were assigned to a value of 0 pg/mL, while those exceeding the upper LOD were capped at the upper LOD value.

S.4 Western Blot

THP-1-derived macrophages, stimulated as described, were washed with PBS and lysed in RIPA buffer supplemented with EDTA-free protease inhibitors (1:10 dilution). Lysates were incubated on ice for 30 minutes, briefly sonicated, and centrifuged at 10,000 × g for 10 minutes at 4 °C. Protein concentration in the supernatant was determined using the Bradford assay (RotiQuant Universal, Carl Roth), with BSA standards ranging from 0–2 µg/µL. Absorbance was measured at 492–503 nm using ClarioStar Plus BMG microplate reader Labtech, Ortenberg, Germany. Twenty micrograms of protein per sample were mixed with DTT-containing loading buffer (1:3), denatured at 95 °C for 2 minutes, and resolved on TGX SDS-PAGE gels (Bio-Rad). Electrophoresis was performed at 200 V for 35 minutes. Proteins were transferred to PVDF membranes using a semi-dry transfer system (Trans-Blot Turbo, Bio-Rad) at 2.5 A, 25 V for 3 minutes. Membranes were blocked in 10% milk/TBST for 1 hour and incubated overnight at 4 °C with primary antibodies for cleaved Caspase-1 (#3866 Cell Signaling Technology, Cambridge UK) or Gasdermin D (#36425 Cell Signaling Technology, Cambridge UK) each diluted in 10% milk/TBST. After washing, HRP-conjugated secondary antibodies were applied for 2 hours at room temperature (mouse-anti-rabbit, 1:15,000, 111-035-144 Jackson ImmunoResearch, Europe Ltd. Cambride, UK). Signals were detected by chemiluminescence after substrate incubation according to the manufacturer's instructions and visualized using a ChemiDoc™ (MP) imaging system (Bio-Rad Laboratories GmbH (Feldkirchen, Germany). To quantify target protein expression relative to β-actin, membranes were stripped with stripping buffer (RotiR-Free Stripping Puffer 2.0) for 30 minutes, re-blocked, and incubated overnight with anti-ACTB antibody (1:15,000, MAB1501R Merck Millipore, Massachusetts, USA), followed by anti-mouse HRP-conjugated secondary antibody (1:15,000, 715-545-150, Jackson ImmunoResearch Europe Ltd., Cambridge, UK). Bands were detected as before. Band intensities were analyzed using ImageLab software (Bio-Rad). After optimizing contrast, bands were manually marked, and adjusted volumes were calculated. Target protein signals were normalized to β-actin and expressed relative to control conditions. Results are depicted in Figure S2)

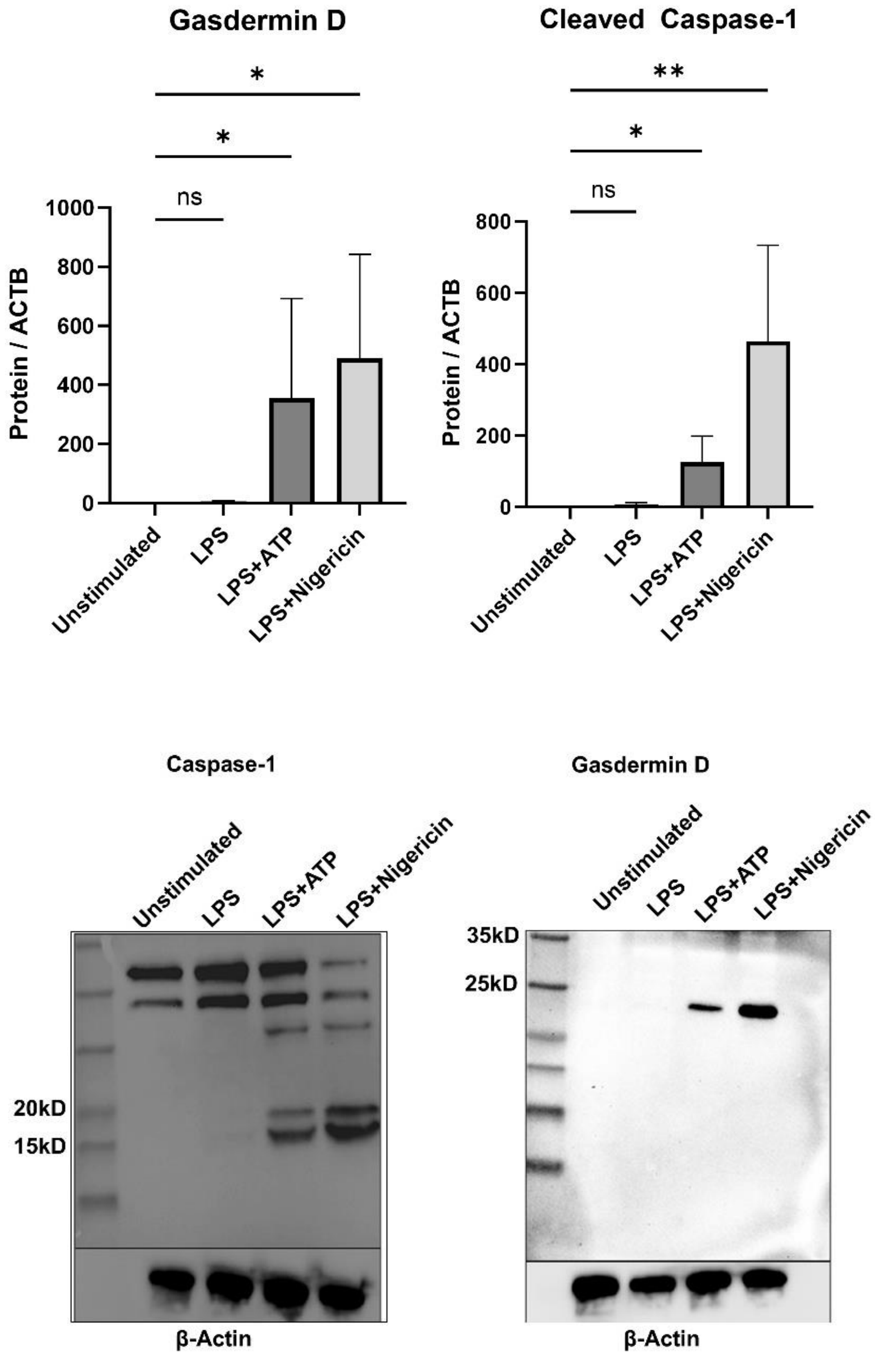

Figure S2: Quantification of Western Blot of Cleaved Caspase-1 and Gasdermin D: Upper panel with protein levels normalized to β-Actin; Mean + SEM., Kruskal-Wallis Test +Dunns Test, n= 5, ns = non-significant, (*) = p < 0,05, (**) p < 0,01. Lower panel: Representative Western-Blot.

S.5 PLA-Assay Validation in THP-1 Macrophages

To verify assay specificity before applying it to patient samples, the PLA was first validated in THP-1–derived macrophages under canonical NLRP3 activation conditions. Upon stimulation with LPS and either ATP or Nigericin, PLA signal intensity increased markedly compared to unstimulated or LPS-only controls, indicating robust formation of the NLRP3–Caspase-1 complex. Quantification showed a 5–6-fold increase in PLA signal in both LPS + ATP and LPS + Nigericin groups ($p < 0.05$), while LPS alone had no significant effect. PLA signal correlated strongly with caspase-1 activity ($r_s = 0.689$, $p < 0.001$), IL-1β ($r_s = 0.693$, $p < 0.001$), and IL-18 levels ($r_s = 0.667$, $p < 0.001$). At the protein level, PLA intensity showed a strong correlation with cleaved caspase-1 ($r_s = 0.815$, $p < 0.001$) and a moderate correlation with cleaved gasdermin D ($r_s = 0.484$, $p < 0.001$). These findings confirm that the PLA specifically and reliably detects functional NLRP3-Caspase-1 inflammasome activation in-vitro (Figure S3).

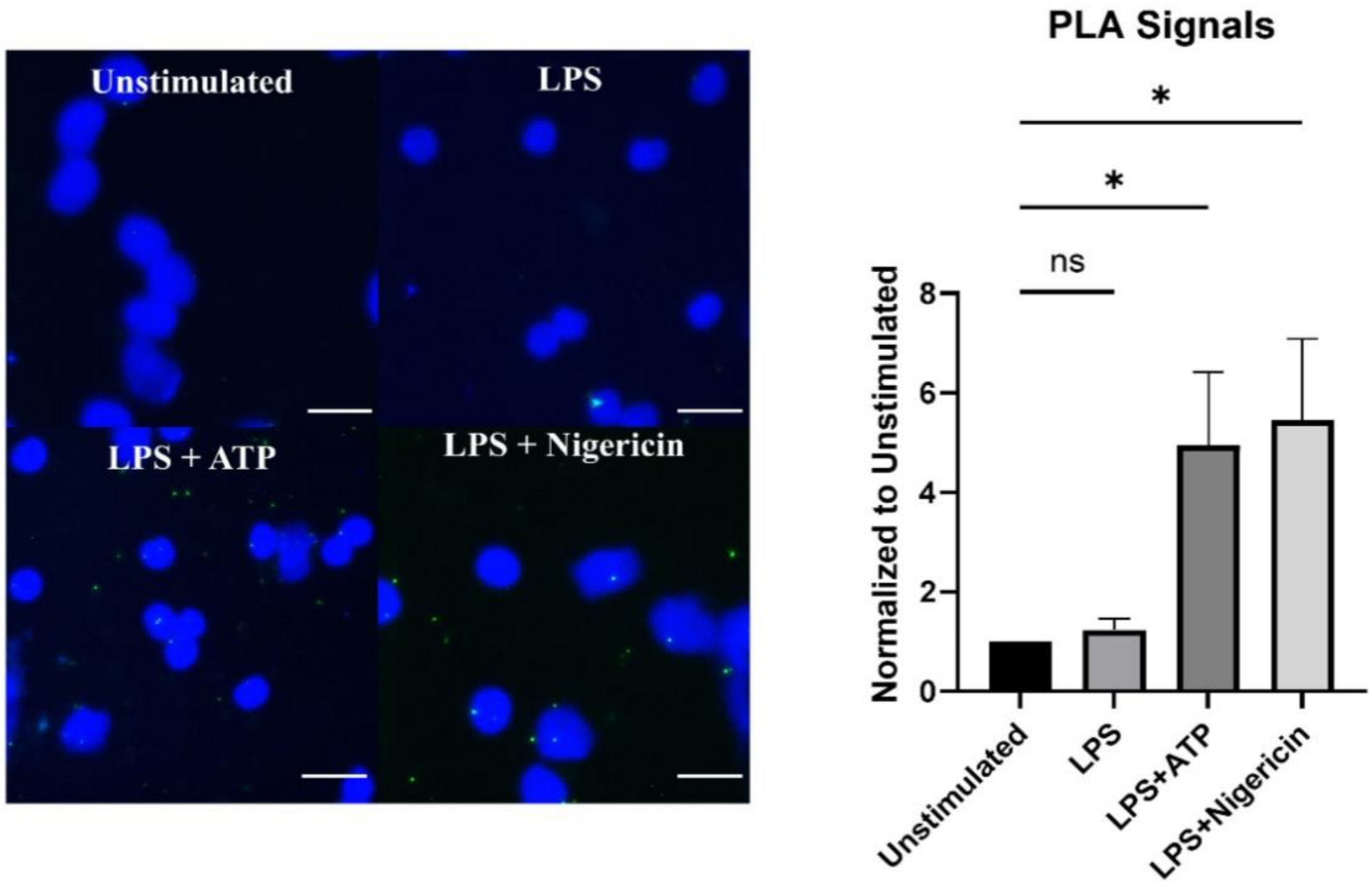

Figure S3: NLRP3-Caspase-1 PLA of stimulated THP-1 Macrophages: Representative fluorescence microscopy images (left) and quantitative analysis (right) of PLA signal intensity under different stimulation conditions. Cells were primed with LPS (1 µg/mL, 3 h) and stimulated with either ATP (5 mM, 1 h) or Nigericin (10 µM, 1 h). Green PLA dots indicate NLRP3–Caspase-1 complex formation; nuclei are stained with DAPI (blue). Scale bar: 10 µm. Quantification (normalized to unstimulated control) shows significantly increased PLA signal upon LPS + ATP and LPS + Nigericin stimulation (mean ± SD; *$p < 0.05$, ns = not significant; Kruskal–Wallis with Dunn's post hoc test; n = 5 independent experiments)
